# Selective Destruction of Soluble Polyurethaneimide as Novel Approach for Fabrication of Insoluble Polyimide Films

**DOI:** 10.3390/polym14194130

**Published:** 2022-10-02

**Authors:** Andrey L. Didenko, Aleksey Gennad’evich Ivanov, Valentina E. Smirnova, Gleb V. Vaganov, Tatyana S. Anokhina, Ilya L. Borisov, Vladimir V. Volkov, Alexey V. Volkov, Vladislav V. Kudryavtsev

**Affiliations:** 1 A.V. Topchiev Institute of Petrochemical Synthesis, Russian Academy of Sciences, Leninsky pr., 29, 119991 Moscow, Russia; 2 Institute of Macromolecular Compounds, Russian Academy of Sciences, Bolshoy pr. V.O., 31, 199004 St. Petersburg, Russia

**Keywords:** insoluble polyimides, soluble poly(urethane-imide), selective destruction, thermolysis, hydrolysis, poly-(4,4′oxydiphenylene)pyromellitimide, film

## Abstract

Polymeric coatings and membranes with extended stability toward a wide range of organic solvents are practical for application in harsh environments; on the other hand, such stability makes their processing quite difficult. In this work, we propose a novel method for the fabrication of films based on non-soluble polymers. The film is made from the solution of block copolymer containing both soluble and insoluble blocks followed by selective decomposition of soluble blocks. To prove this concept, we synthesized copolymer [(imide)_n_-(polyurethane)]_m_, in which the imide blocks were combined with polyurethane blocks based on polycaprolactone. By selective hydrolysis of urethane blocks in the presence of acid, it was possible to obtain the insoluble polyimide film for the first time. It was shown that the combination of thermal and acid treatment allowed almost complete removal of urethane blocks from the initial copolymer chains. IR spectroscopy, TGA, DSC and DMA methods were used to study the evaluation of the structure and properties of polymeric material as a result of thermal oxidation and hydrolysis by acid. It was shown that the polymeric films obtained by controlled decomposition were not soluble in aprotic solvent, such as dimethylformamide, n-methylpyrrolidone and dimethyl sulfoxide, and showed very close similarity to the homopolymer consisting of the same imide monomer, poly-(4,4′oxydiphenylene)pyromellitimide, confirming the feasibility of the proposed concept and its perspectives for fabrication of organic solvent-resistant membranes.

## 1. Introduction

Copoly(urethane-imide)s combine the properties of two important classes of polymers for technology: polyurethanes and polyimides. Almost all types of industrial materials (coatings, films, fibers, plastic masses, foams, etc.) capable of operating in a variety of technical devices for thousands of hours at 300 °C and, importantly, at high levels of radiation are produced on the basis of polyimides [1,2,3,4,5,6,7,8,9,10,11,12,13,14]. Poly-(4,4′-oxydiphenylene) pyromellitimide is widely known because it is processed in a large volume into an electrical insulating film [15]. Multiblock (segmental) copoly(urethane-imide)s, which have been actively developed in recent decades, are of potential interest as a new generation of high-temperature, high-strength elastomers [16,17,18,19,20,21]. The physical properties of copoly(urethane-imide)s are mainly determined by the presence of poorly thermodynamically compatible imide and urethane elements in the macrochains. Statistical copoly(urethane-imide)s with increased content of imide units have a practical interest due to their ability to transition from polyimide thermoplastics to novel copoly(urethane-imide) thermoplastic elastomers [22,23]. Polyimides are considered robust materials for the fabrication of different membranes for gas and liquid separation [6,8,24,25,26,27,28], especially for the development of high-performance nanofiltration membranes for water treatment or organic solvent regeneration in the pharmaceutical industry [29,30,31,32,33].

In addition to homopolymers, copolymers containing various imides segments are also drawing interest; particularly, copoly(urethane-imide)s can be successfully used as pervaporation membranes for the separation of aromatic hydrocarbons in mixtures with aliphatic and alicyclic hydrocarbons [34]. However, imide and urethane units possess significant differences in thermal and chemical resistance [22,23]. For instance, different approaches to the decomposition of urethane-based copolymers were proposed in the literature, including hydrolysis, glycolysis and aminolysis [35,36]. At the same time, it is well known that polyimides are extremely stable in acidic media but degrade in the presence of nucleophilic agents. It seems reasonable to use the differences in the reactivity of imide and urethane links with respect to thermolysis, as well as hydrolysis in acidic media to impart a nano- and microporous structure to polymer systems such as [(imide)_n_-(urethane)]_m_ due to controlled destruction of urethane links.

**Scheme 1:**
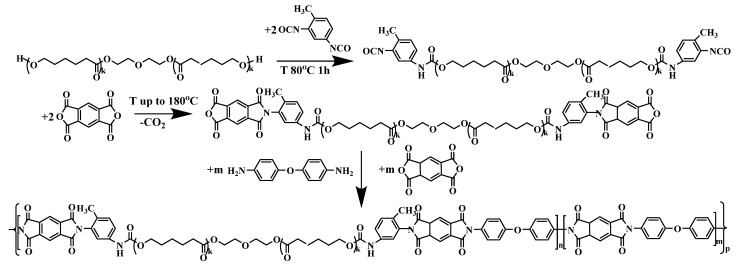
Synthesis of copolymer of [(imide)n-(urethane)]m type.

The aim of this work is to show the possibility of selective destruction of urethane links in copoly(urethane-imide)s with increased imide link content without destroying the integrity of the original samples. The kinetics of changes in the deformation–strength properties of a number of polyimides in aqueous–alkaline media was investigated in recent work [37]. A complex architecture copolymer of [(imide)_n_-(urethane)]_m_ type, including units of poly-(4,4′-oxydiphenylene) pyromellitimide and units of bisurethane obtained on the basis of polycaprolactone and 2,4-toluene diisocyanate, was chosen in this study. This choice makes it possible to predict the stability in corrosive environments of poly-(4,4′-oxydiphenylene)pyromellitimide modified by the inclusion of urethane links in its structure. It should be noted that poly-(4,4′-oxydiphenylene)pyromellitimide in weakly concentrated alkaline solutions decomposes to monomers, which were used for the recovery of production wastes [2,3,4,5] (Scheme 1). It should be noted that unlike polyimides, polyurethanes undergo destruction in acid solutions [35,37,38].

## 2. Materials and Methods

### 2.1. Materials

The copoly(urethane-imide) chosen as the object of study was obtained according to the synthesis method outlined in our initial studies [39,40,41,42,43,44,45]. At that, pyromellitic anhydride with a melting point (T_m_)~283–286 °C, Sigma-Aldrich Co., LLC, Burlington, MA, USA; polycaprolactone with molecular weight Mn = 2000, Sigma-Aldrich Co. LLC with melting point (T_m_)~50 °C; 2,4- toluene diisocyanate with melting point (T_m_)~20–22 °C, Sigma-Aldrich Co., LLC; 4,4′-diaminodiphenyl ether with melting point (T_m_)~188–192 °C, Sigma-Aldrich Co., LLC. were used as monomers. *N,N*-dimethylacetamide, Vecton Co., LLC, Russia, Saint Petersburg was used as a solvent.

### 2.2. Synthesis Method

The method used to synthesize the target copolymer assumes copolycondensation of 4,4′-diaminodiphenyl ether in solution in *N,N*-dimethylacetamide with pyromellitic anhydride and macromonomer with end anhydride groups, which is obtained based on polycaprolactone, 2,4-toluene diisocyanate and pyromellitic anhydride. 

### 2.3. Synthesis of an Initial Macromonomer with Urethane Groups in the Chain

In a three-neck flask equipped with an overhead stirrer and an argon feed and output tube, 5.0 g (2.5 mmol) of polycaprolactanediol and 0.87 g (5.0 mmol) of toluene-2,4-diisocyanate were loaded. The mixture was heated in an oil bath to 80 °C and incubated for 1 h. Then, 1.1 g (5.0 mmol) of pyromellitic dianhydride as a finely ground powder was added. The mixture was heated to 180 °C and stirred for two hours until homogenization of the resulting melt and cessation of bubbling of carbon dioxide formed during the reaction. The reaction mixture was cooled to 160 °C and 23 mL of *N,N*-dimethylacetamide was added there to dissolve the resulting product. The solution concentration on macromonomer was 23 wt%. The macromonomer was not isolated from the reaction solution.

### 2.4. Synthesis of Copoly(Urethane-Imide) Prepolymer

In a flask containing the macromonomer solution prepared as shown above, 11.0 g (55.0 mmol) of 4,4′-diaminodiphenyl ether and 12.0 g (55.0 mmol) of pyromellitic anhydride were loaded, and then 50 mL of *N,N*-dimethylacetamide was immediately added. After dissolution of the monomers, the reaction mixture was stirred intensively for 4 h under argon current at room temperature to complete the diamine polyacylation reaction. We obtained the prepolymer (copoly(urethane amide acid) in solution in *N,N*-dimethylacetamide. The solution with a concentration of 30 wt% of prepolymer was passed through a Schott filter, degassed under vacuum, and then the films were cast from it.

### 2.5. Obtaining Film Samples of Copoly(Urethane-Imide)

The obtained copoly(urethane-imide) prepolymer solution was cast on the hydrophobized glass plates. Films were dried in the thermostat in the following temperature conditions: 12 h at 80 °C, 1 h at 100 °C, 1 h at 120 °C. For copoly(urethane-amide acid) thermal conversion to the target copoly(urethane-imide), the samples were heated for 1 h at 140 °C and for 2 h at 170 °C. After that, the films could be removed from the plates by immersion in hot water. The thickness of the films was about 100 μm. Subsequent thermolysis of the copolymer was performed using samples left on the plates.

### 2.6. The Synthesized Polymer Corresponds to the Structural Formula

**From Scheme 1**.


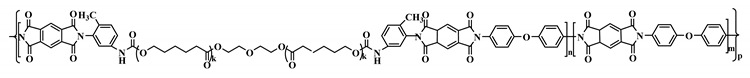
 where *n* = 1, m = 10.

NMR ^1^H prepolymer (DMSO-d_6_) δ, figures: 9.91, 9.62, 9.12, 8.87, 8.38, 7.98–6.55, 3.99, 3.66, 2.29, 2.15, 2.11, 2.08, 1.54, 1.31

The intrinsic viscosity of the prepolymer was [η]= 1.1 × 100 cm^3^ g^−1.^

**Scheme 2:**
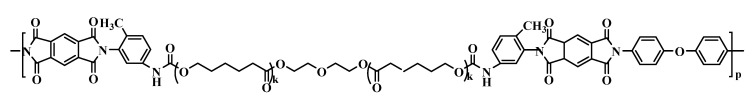
The formula of multiblock (segmental) copoly(urethane-imide).

As a comparison object, a multiblock (segmental) copoly(urethane-imide) (Scheme 2) which contained one aromatic and one aliphatic block in a repeating link was synthesized. Synthesis of multiblock copoly (urethane-imide) was carried out under working conditions [39,40,41,42]. The molar ratio of the initial monomers of pyromethyl anhydride, diamine and caprolactone was 2:1:1.

NMR ^1^H prepolymer (DMSO-d_6_) δ, figures: 9.91, 9.62, 9.12, 8.87, 8.38, 7.96–6.52, 3.99, 3.66, 2.28, 2.15, 2.11, 2.08, 1.55, 1.30

The intrinsic viscosity of the prepolymer was [η] = 0.89 × 100 cm^3^ g^−1.^

### 2.7. Thermolysis of Copoly(Urethane-Imide)

The copoly(urethane-imide) samples mounted on the glass plates were placed in a SNOL 67/350 programmable thermal oven with natural air convection. The annealing temperature was varied from 170 °C to 350 °C with durations ranging from 0.5 h to 2 h. After annealing, the films were removed from the plates after boiling in water and examined.

### 2.8. Hydrolysis of Thermalized Samples of Copoly(Urethane-Imide)

After completion of thermal annealing, samples of self-bearing copoly(urethane-imide) films were placed in the baths containing aqueous solutions of acids. The baths were filled with mixtures of acetic acid (Vecton Co., LLC, Saint Petersburg, Russia) and concentrated hydrochloric acid (Vecton Co., LLC, Saint Petersburg, Russia) taken in ratios of 90:10 vol% and 10 to 90 vol%, respectively; acetic acid (Vecton Co. LLC, Russia) and ethyl alcohol (Vecton Co., LLC, Russia) taken in a ratio of 90:10 vol%; acetic acid (Vecton Co., LLC, Saint Petersburg, Russia) and isopropyl alcohol (Vecton Co., LLC, Saint Petersburg, Russia) taken in a ratio of 90:10 vol%; trifluoroacetic acid (Vecton Co., LLC, Russia Saint Petersburg) and water taken in a ratio of 50:50 vol%. The samples were soaked in the selected bath at room temperature for 1 to 2 days, removed from the bath and rinsed with distilled water until the rinse water showed a neutral reaction. Then, the films were dried in the open air and examined.

### 2.9. Methods of Research

IR spectra of the films were recorded on a Fourier spectrometer “IRAffinity-1S” (Shimadzu, Kyoto, Japan) in the mid-IR region (700–4000 cm^−1^) using a MIRacle microadapter (PIKE Technologies, Madison, Wisconsin, USA) of single attenuated total internal reflection (ATIR).

Then, 1H NMR spectra were recorded in deuterated DMSO (DMSO-d_6_) on a high-resolution AVANCE II-500 WB NMR spectrometer (Bruker, Billerica, MA, USA) at an operating frequency of 500 MHz at room temperature, measurement range 1–10 ppm. 

The characteristic viscosity was measured on the Ubbelode viscometer of the reverse flow of liquids. Sample volume: 11 mL. Accuracy: =0.2%. The viscosity was measured at a temperature of 20 °C in *N,N*-dimethylacetamide.

Thermogravimetric analysis (TGA) curves were taken on a TG 209 F1 (NETZSCH, Zelb, Germany) in the temperature range from 30 °C to 800 °C at a heating rate of 10 °C/min in an inert medium (argon). Weight of the samples was 2–3 mg.

Temperature changes of glass transition and melting of film copolymer samples were determined by differential scanning calorimetry (DSC) on a DSC 204 F1 (NETZSCH, Zelb, Germany) at a heating rate of 10 °C/min in the temperature range from −60 °C to 300 °C in an inert medium (argon). Weight of the samples was 4–5 mg. As applied to the copolymers under study, the DSC method characterizes melting processes of microcrystalline phase formed by aliphatic polyether units.

The temperature dependences of the dynamic values: storage modulus E’, loss modulus E’’ and the tangent of the mechanical loss angle tan δ of the film samples were obtained by dynamic mechanical analysis (DMA) on a DMA 242 C machine (NETZSCH, Zelb, Germany). Measurements were taken at a frequency of 1 Hz, a temperature rise rate of 5 °C/min and a deformation amplitude of 0.1%. The glass transition temperature of the film samples was determined by the temperature of maximum tan δ.

The deformation–strength characteristics of the films were determined at room temperature in the mode of uniaxial tension on samples in the form of strips 2 mm wide with the length of the working part of 25 mm. The tests were carried out on an Instron 5940 universal tensile testing machine (Instron, Switzerland). Tensile testing of the film samples was carried out at a speed of 10 mm/min.

## 3. Results

The target copoly(urethane-imide) was obtained by copolycondensation in a solution in *N,N*-dimethylacetamide of one monomer—4,4′-diaminodiphenyl ether with two comonomers—pyromellitic anhydride and macromonomer specially prepared on the basis of polycaprolactone, which contained urethane groups in the molecular chain and had end anhydride groups. The formation of the stated macromonomer took place in two stages. At the first stage, during the interaction of the end hydroxyl groups of polycaprolactone with a double molar excess of 2,4-toluene diisocyanate, an intermediate macromonomer with urethane groups in the macrochain and end isocyanate groups was formed. In the next stage, the interaction of the end isocyanate groups of the intermediate macromonomer with a double molecular excess of pyromellitic anhydride resulted in the formation of a macromonomer with end anhydride groups and urethane groups in the chain. The formation of intermediate and final macromonomers proceeded by the mechanisms of urethane chemistry [16,22,23], and the polycondensation process of copoly(urethane-imide) formation followed the mechanism of acid anhydride acylation of amines used in polyimide chemistry [1,2,3].

According to the above structural formula, the synthesized copoly(urethane-imide) contained polycaprolactone links framed by urethane bridge groups. It can be a priori stated that aliphatic links of polycaprolactone are less resistant to thermal–oxidative destruction compared to pyromellitimide aromatic links, and urethane bonds are not stable in aggressive acidic environments compared to imide bonds. In view of the aforesaid, it seems to us possible to carry out selective destruction of the urethane links of the synthesized copoly(urethane-imide) by thermolysis or thermolysis in combination with destruction in acidic media.

### 3.1. TGA Studies of Synthesized Copolymers

**Figure 1:**
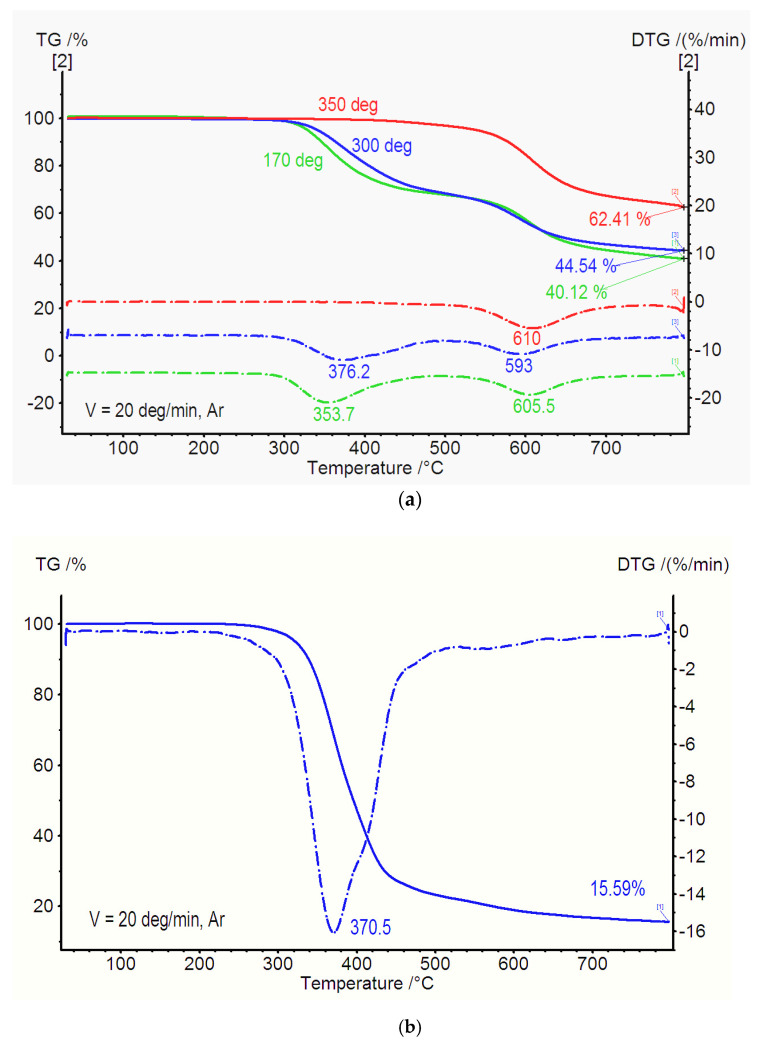

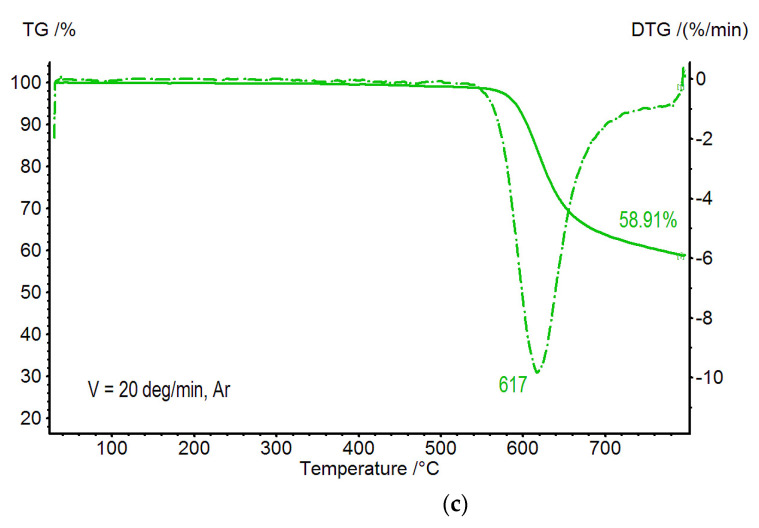
TGA and DTG curves: (**a**) copoly(urethane-imide) [(imide)_11_-(polyurethane)]_m_ thermalized for 30 min at temperatures: 170 °C (green curves), 300 °C (blue curves), 350 °C (red curves); (**b**) multiblock segmental copolyurethane-imide (Scheme 2); (**c**) laboratory sample of poly-(4,4′-oxydiphenylene) pyromellitimide. Note: The heat resistance indices τ_5_ and τ_10_ are the corresponding 5 and 10% weight loss of the samples.

The results of the research of thermal resistance, heat resistance and mechanical properties of the synthesized copolymer film samples after heating them in the air for 30 min at temperatures of 170 °C, 300 °C and 350 °C, respectively, are presented below. Figure 1 shows the curves of thermogravimetric analysis (TGA) and differential thermogravimetric analysis (DTG), from which the values of τ_5_ and τ_10_ temperature indices of the heat resistance of heated samples were determined. The indices τ_5_ and τ_10_ denote temperatures corresponding to 5% and 10% mass loss of the sample. As follows from the DTG curves (Figure 1a), the indices τ_5_ increased with increasing temperature of thermolysis of the sample: 353 °C in case of heating at 170 °C, 376 °C in case of heating at 300 °C and 610 °C in case of heating at 350 °C. In the latter case, the value of index τ_5_ approached the value τ_5_ = 617 °C, typical for poly-(4,4′-oxydiphenylene) pyromellitimide (Figure 1b). 

It is noteworthy that on the TGA curves for the samples heated at 170 and 300 °C, inflections were observed in the temperature ranges of 300–430 °C and 550–650 °C. At the same time, for the sample heated at 350 °C, one inflection in the range of 550–650 °C was observed. As mentioned above, the inflection in the range of 550–650 °C corresponds to the destruction of poly-(4,4′-oxydiphenylene) pyromellitimide. As seen in Figure 1c, a single inflection on the TGA curve in the range of 300–430 °C was observed for the multiblock segmental copolyurethane-imide (Scheme 2). Thus, we can conclude that during the destruction of copoly(urethane-imide) of [(imide)_11_-(polyurethane)]_m_ type the inflection in the range of 300–430 °C on the TGA diagram corresponded to the destruction of an aliphatic block, and the inflection in the range of 550–650 °C corresponded to the destruction of a polyimide block. That is, during thermolysis of the studied copoly(urethane-imide) at the temperature range of 300–350 °C, selective destruction of urethane units took place.

### 3.2. DSC Studies of Synthesized Copolymers

**Figure 2:**
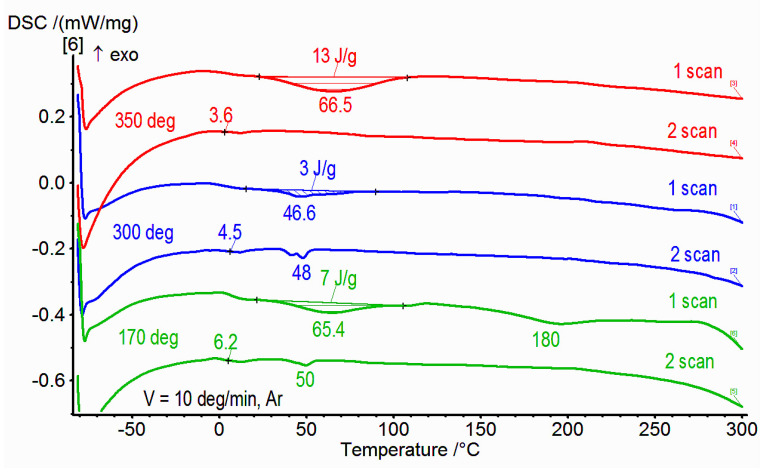
DSC curves of copoly(urethane-imide) samples thermalized for 30 min at temperatures: 170 °C (green curves), 300 °C (blue curves) and 350 °C (red curves).

As follows from the analysis of the thermalized samples of synthesized copoly(urethane-imide) (Figure 2) by the differential scanning calorimetry (DSC) method, the thermograms of the first and second samples heated at 170 °C showed glass transition (6.2 °C) of aliphatic polycaprolactone links (blocks). The curves of the first heating showed endotherms which should be attributed to the melting of the aliphatic crystal phase: at 64 °C (ΔH = 7 J/g) and glass transition of noncyclic amic acid links (180 °C). In the case of the sample heated at 350 °C, at 66 °C (ΔH = 13 J/g) a peak corresponding to the melting of the aliphatic crystal phase was observed in the first scanning thermal diagram. In accordance with the ideas about the properties of polyurethanes [16,22,23], in copoly(urethane-imide)s flexible aliphatic blocks and rigid aromatic blocks were thermodynamically poorly compatible and formed separate microphases. 

It follows from the DSC experiment that in the studied copoly (urethane-imide) there was interpenetration of blocks of aromatic and alifatic phases, which increased the thermal stability of the urethane blocks in the mixed phase with imide blocks. This structure was present in all the examined samples heated in the temperature range of 170–350 °C. However, taking into consideration the low thermal melting effect of mixed structures that we observed (3–13 J/g), it can be stated that the share of such mixed structure in the copolymer was insignificant. This is also confirmed by the significant loss of polymer mass during annealing from 170 to 350 °C (23.2% wt.), while the total aliphatic block content in the studied copoly(urethane-imide) was 25.1% wt. 

### 3.3. DMA Studies and Mechanical Properties of Thermalized Samples

**Figure 3:**
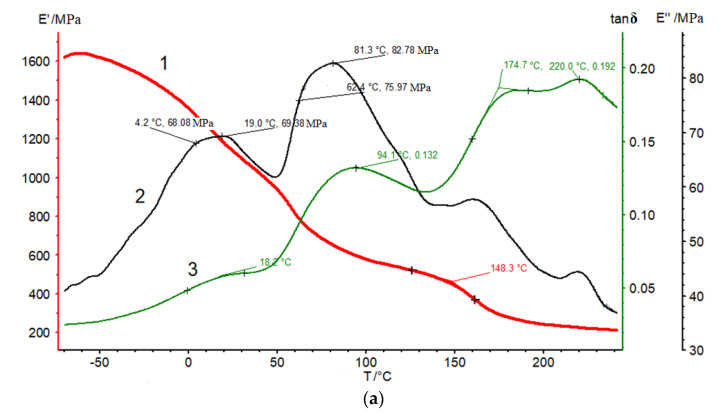

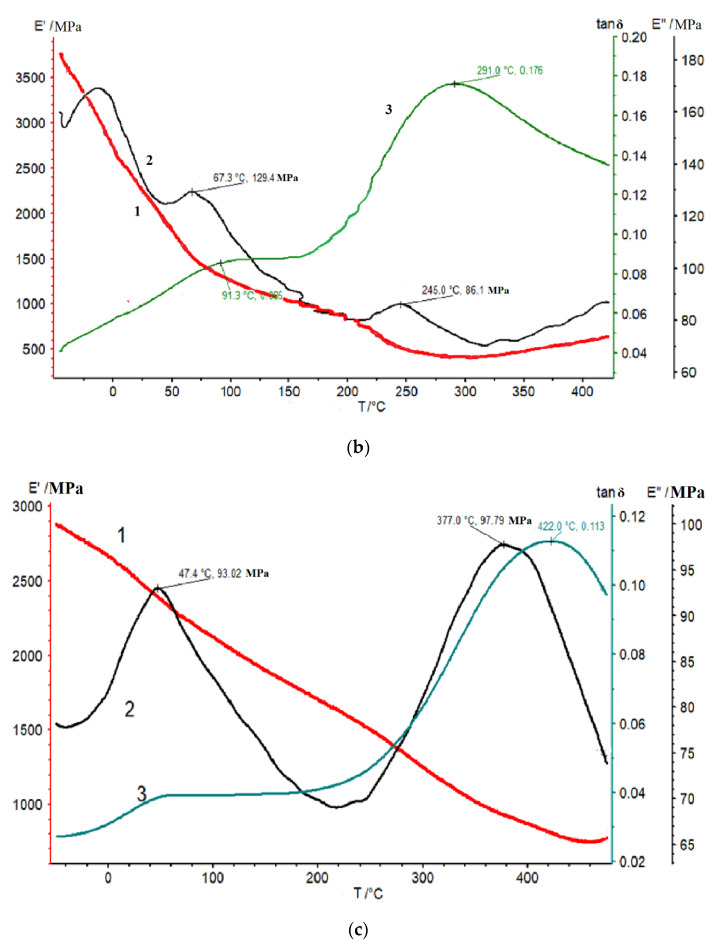
DMA curves of copoly(urethane-imide) samples thermalized for 30 min at temperatures: 170 °C (**a**); 300 °C (**b**); 350 °C (**c**). Temperature dependencies E’ (red curve), E’’ (black curve) and tan δ (blue curve).

The presence of mixed aliphatic-aromatic structures in the thermolysis products of the studied copoly(urethane-imide) was indicated by the results of experiments carried out by means of dynamic mechanical analysis (DMA) (Figure 3). It should be noted that according to DMA data, the glass transition temperature of the aliphatic phase of multiblock (segmental) copoly(urethane-imide) obtained on the basis of polycaprolactone (Mn 2000), pyromellitic anhydride and 4,4′-diaminodiphenyl ether was T_g_ = −33 °C.

DMA curves of the sample heated at 170 °C are shown in Figure 3a. Because of the increased content of aromatic imide blocks in the polymer system, the glass transition temperature of the aliphatic phase shifted to the region of higher temperatures and had the value T_g_ = 18 °C. The maximums on the temperature dependencies E’ (64 °C–80 °C) and tan δ (94 °C) corresponded to the aliphatic crystal phase melting area, the maximums on the dependencies E’ (162 °C) and tan δ (181 °C) corresponded to the glass transition temperature of the uncyclized amic acid fragments. Finally, the maximums on the dependencies E’’ (219 °C) and tan δ (220 °C) were responsible for the glass transition of the aromatic phase. The curves in Figure 3b, corresponding to the sample heated at 300 °C, generally indicate an increase in the content of aromatic blocks in the polymer system due to the tightening of temperature conditions of thermolysis. In the case of the sample heated at 350 °C (Figure 3c), there was a microphase separation of the rigid aromatic aliphatic phase and “residuals” of the flexible aliphatic phase. We can state with satisfaction the mutual confirmation of the results of experiments on thermolysis of the studied copoly(urethane-imide), carried out using TGA and DMA methods: selective “removal” of aliphatic blocks from the polymer system as a result of thermolysis of copoly(urethane-imide). 

Thermolysis of copoly(urethane-imide) can be of practical importance if thermolysis does not significantly reduce the deformation–strength properties of the tested films compared to the original polyimide, in our case compared to poly-(4,4′-oxydiphenylene)pyromellitimide.

It is noteworthy that as the thermolysis temperature of the copolymer increased from 170 °C to 350 °C (i.e., the relative content of aromatic imide blocks increased while the content of aliphatic urethane blocks in the polymer decreased), the mechanical properties of the thermolysis products approached the characteristics of poly-(4,4′-oxydiphenylene)pyromellitimide. According to the data shown in Table 1, the studied copoly(urethane-imide) thermalized at 350 °C was close to the laboratory sample of poly-(4,4′-oxydiphenylene)pyromellitimide by mechanical properties. It should be noted that the films heated at 300 and 350 °C did not dissolve and had stable mechanical properties in such aprotic solvents as dimethylformamide, n-methylpyrrolidone and dimethyl sulfoxide. Thus, the developed self-supported thermalized films have great opportunities to obtain mechanically strong polymer membranes based on them, which are capable of operating at elevated temperatures and pressures. However, since poly-(4,4′-oxydiphenylene)pyromellitimide is designed for long-term operation at 300 °C, it is preferable to perform thermolysis at temperatures under 300 °C.

In this regard, the process of selective destruction of copoly(urethane-imide) combines thermolysis and hydrolysis of urethane blocks in the copolymer. In the presented work, we performed experiments in which the film samples subjected to thermolysis at 300 °C for 30 min were kept in a bath containing a mixture of hydrochloric and acetic acids for 24 and 48 h. At the same time, the polymer product was enriched with imide blocks by removing the residual amount of urethane blocks. If the destruction selectivity during thermolysis is determined by differences in the thermal stability of aromatic and aliphatic blocks, then in the case of hydrolysis, the hydrolytic stability of the imide bonds and the tendency to split urethane bonds, which are characteristic of acidic media, become important [1,2,3,22,23].

**Figure 4:**
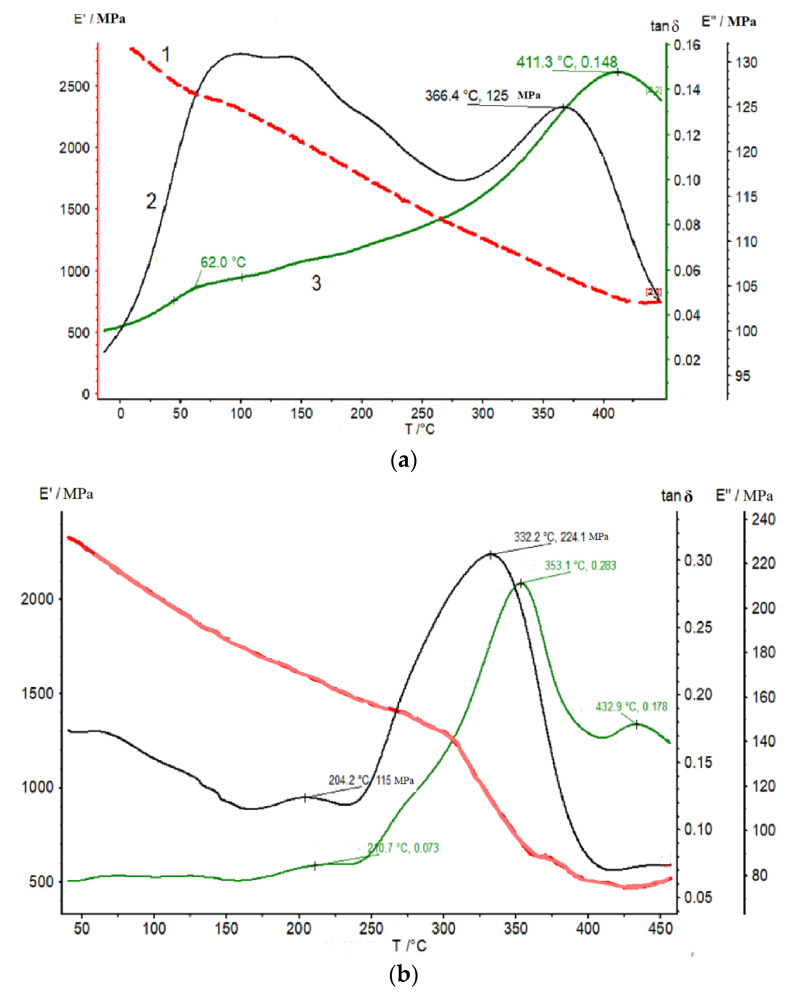
DMA curves: (**a**) film of poly(urethane-imide) thermalized at 300 °C for 30 min after soaking it for 48 h at 20 °C in a mixture of hydrochloric acid (90% vol.) and acetic acid (10% vol.); (**b**) laboratory sample of poly(4,4′-oxydiphenylene)pyromellitimide film. Temperature dependence of accumulation modulus E’ (red curve), loss modulus (blue curve) and tan δ (green curve).

DMA thermograms of the obtained sample (Figure 4a) show that the imide blocks content increased in the final polymer because of soaking of the prethermalized film in an acidic aggressive medium.

Comparison of the DMA results of polymer samples subjected to thermalization (Figure 3b) and thermalization with subsequent soaking in an acidic aggressive environment (Figure 4a) shows that the result of samples soaking in an acidic aggressive medium is the practical absence on the DMA curves of inflections corresponding to the temperature transitions of aliphatic blocks. Thus, it is reasonable to assume that the thermolysis process of the copoly(urethane-imide) decelerates due to spatial difficulties in the movement (screening) of aliphatic links in the mixed aliphatic-aromatic phases (Figure 3). It is probable that hydrolytic decomposition of urethane bonds and subsequent removal of aliphatic fragments into the reaction medium takes place in acidic aggressive media.

The data presented in Table 2 correspond to the initial samples obtained by thermolysis of the copoly(urethane-imide) for 30 min at 170 °C (1) and 300 °C (2), respectively, and then soaked in the baths containing mixtures of hydrochloric acid (90% vol) and acetic acid (10% vol) for 24 h and 48 h, respectively.

As evident from Table 2, the examined films were characterized by a high level of deformation–strength properties. At the same time, the sample prepared as a result of soaking in an aggressive medium for 48 h had properties close to the laboratory sample of poly-(4,4′-oxydiphenylene) pyromellitimide, given for reference. Thus, the use of aggressive acidic media is prospective for the selective destruction of urethane links in copoly(urethane-imide) chains with increased content of imide links.

### 3.4. Comparative Study by IR Spectroscopy of the Obtained Copolymers

**Figure 5:**
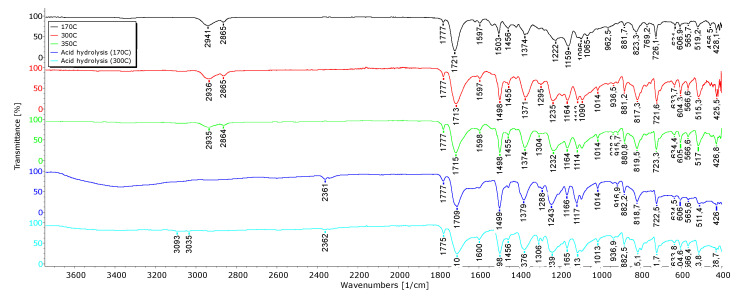
IR spectrum of the copoly(urethane-imide) film thermalized at 170 °C (black curve) for 30 min; film samples thermalized for 30 min: at 300 °C (red curve) and at 350 °C (green curve); film samples soaked in a mixture of concentrated hydrochloric acid (90% vol) and acetic acid (10% vol) for 24 h after heating for 30 min: at 170 °C (blue curve) and 300 °C (turquoise curve).

The changes in the physical properties of polymer films reviewed above are determined by changes in the chemical structure of polymers, which are detected by monitoring the process of selective destruction of urethane links in the copoly(urethane-imide) by IR spectroscopy in the frequency range of 4000–400 cm^−1^ [46,47]. Figure 5 shows the IR spectrum of the product of thermolysis of copoly(urethane-imide) carried out at 170 °C for 30 min. In the spectrum range of 3300–3400 cm^−1^, there are bands corresponding to the valence vibrations of the secondary amino groups belonging to urethane groups. The broadened character of these bands indicates the possible participation of urethane groups in the formation of hydrogen bonds [48]. In the range of 3030–3100 cm^−1^, low-intensity bands of the C–H valence vibrations of aromatic diamine radicals and dianhydrides are present in the spectra. The mid-intensity bands of C–H valence vibrations of aliphatic blocks (caprolactone fragments) are found in the range of 2850–2970 cm^−1^. The valence C=O vibrations of the imide cycles are confirmed by the presence of characteristic bands in the range of 1777 cm^−1^ and 1721 cm^−1^ corresponding to asymmetric and symmetric vibrations, respectively, which are overlapped by the C=O vibration band of the ester group of the caprolactone fragments. The valence vibrations of C=O groups in the urethane fragments appear in the 1620 cm^−1^ band which is characteristic for C=O groups involved in the formation of hydrogen bonds [49]. The presence of a band of very low intensity in the range of 1650–1660 cm^−1^ indicates the presence of a residual number of “abnormal” amic acid links in the polymer chains, which is probably due to the incomplete imidization reaction of the prepolymer when producing copoly(urethane-imide). The bands of average intensity located in the range of 1597 cm^−1^ and in the interval of 1534–1456 cm^−1^ correspond to the C–C vibrations of the aromatic rings. A broad band with a maximum in the range of 1159 cm^−1^ corresponds to C–O vibrations of the urethane fragment, the band at 1113 cm^−1^ corresponds to asymmetric valence vibrations of the C–O bond in the ester group, and the band at 1096 cm^−1^ corresponds to vibrations of the ether group of the aliphatic block.

As mentioned above, the aliphatic block is less resistant to thermal–oxidative destruction of copoly(urethane-imide) in comparison with the aromatic block. In fact, as a result of thermolysis of copoly(urethane-imide) at elevated temperatures of 300 °C and 350 °C, there is intensive destruction of the aliphatic block, which is reflected in the IR spectra of thermalized samples (Figure 5). In particular, we notice a practical absence of bands in the range of C–H vibrations of aliphatic groups at 2941 cm^−1^ and 2865 cm^−1^, as well as a significant decrease in the intensity of C=O and C–O vibration bands of the urethane fragment at 1620 cm^−1^ and 1164 cm^−1^, respectively.

Significant changes are observed in the IR spectra of the films soaked in an acidic aggressive medium (mixture of concentrated hydrochloric acid (90% vol) and acetic acid (10% vol) for 24 h) after the completion of heat treatment (Figure 5). In the IR spectra, an absorption increase in the vibration area of O–H groups is detected, which directly indicates the hydrolytic splitting of ester groups. In the case of the sample preheated at 170 °C, the intensity of this band is significantly higher, which is apparently caused by hydrolysis of the residual amic acid links. Changes in the absorption intensity of the C=O and C–O bands of the urethane fragment vibrations at 1620 and 1164 cm^−1^ are more apparent in the case of the sample preheated at 300 °C. It is important that the absorption bands corresponding to vibrations of C–H bonds of the aliphatic block are absent in the spectra, i.e., no urethane blocks are detected in the polymer system.

The spectroscopic study of copoly(urethane-imide)s at different stages of chemical transformations confirms the process of selective destruction of the aliphatic block in their structure.

## 4. Conclusions

The new copoly(urethane-imide) with complex architecture of [(imide)_n_-(polyurethane)]_m_ type was synthesized in which the imide blocks were combined with polycaprolactone-based polyurethane blocks, and the aromatic imide blocks were contained in a ratio prevailing in comparison with the aliphatic polyurethane blocks (75% wt.). In a practical sense, the copoly(urethane-imide) synthesized in this study can be considered as poly-(4,4′-oxydiphenylene)pyromellitimide with polycaprolactone links with urethane end groups introduced in a limited number into its chains. The fundamental possibility of selective destruction of urethane copoly(urethane-imide) blocks in the process of thermal oxidation in air and hydrolysis in acidic media was shown. The IR, DMA and TGA researches of copoly(urethane-imide)s at different stages of chemical transformations confirm the occurrence of selective destruction of the aliphatic block. It was found that with the increase of the thermolysis temperature of the copolymer from 170 °C to 350 °C (accompanied by an increase in the relative content of aromatic imide blocks in the polymer) the mechanical properties of the thermolysis products became close to those of poly-(4,4′-oxydiphenylene)pyromellitimide. Thus, the developed copoly(urethane-imide)s have great potential for obtaining mechanically and chemically stable polymeric films and membranes to be operated at elevated temperatures and pressures.

## Figures and Tables

**Table 1:** Deformation–strength properties of copoly(urethane-imide) films heated for 30 min at the specified temperatures.

No.	Polymer Type and Processing	Polymer Properties		
		E, MPa	σ_t_, MPa	ε_b_, %
1	multiblock (segmental) copoly(urethane-imide) (from Scheme 2)	4 ± 1	33 ± 5	889 ± 176
2	copoly(urethane-imide) (from Scheme 1) 170 °C Thermolysis–2 h	1796 ± 229	113 ± 8	172 ± 15
3	copoly(urethane-imide) (from Scheme 1) 300 °C Thermolysis–0.5 h	1494 ± 96	103 ± 11	106 ± 12
4	copoly(urethane-imide) (from Scheme 1) 350 °C Thermolysis–0.5 h	2452 ± 331	131 ± 16	27 ± 5
5	poly-(4,4′-oxydiphenylene)pyromellitimide	2115 ± 226	122 ± 10	34 ± 4

**Note: Sample No.** 1 of multiblock (segmental) copoly(urethane-imide) obtained from polycaprolactone (Mn 2000), pyromellitic anhydride and 4,4′-diaminodiphenyl ether is given for reference. Sample No. 5 of laboratory poly-(4,4′-oxydiphenylene)pyromellitimide is given for reference.

**Table 2:** Deformation–strength properties of polymer films obtained as a result of selective destruction of urethane blocks in copoly(urethane-imide) in the processes of thermolysis and subsequent hydrolysis of initial samples.

No.	Polymer Type and Processing	Polymer Properties		
		E, MPa	σ_t_, MPa	ε_b_, %
2	copoly(urethane-imide) (from Scheme 1) 170 °C Thermolysis–2 h–hydrolysis 24 h	1038 ± 98	117 ± 10	137 ± 12
3	copoly(urethane-imide) (from Scheme 1) 300 °C Thermolysis–0,5 h–hydrolysis 48 h	1870 ± 150	119 ± 11	72 ± 9
5	poly-(4,4′-oxydiphenylene)pyromellitimide	2115 ± 226	121 ± 10	34 ± 4

## List of Symbols and Acronyms

TGA	thermogravimetric analysis;
DTG	differential thermogravimetric analysis;
DSC	differential scanning calorimetry;
DMA	dynamic mechanical analysis;
NMR ^1^H	nuclear magnetic resonance spectra;
DMSO-d_6_	deuterated dimethyl sulfoxide;
IR	infrared spectra;
*η*	viscosity, cm^3^ g^−1.^;
*τ_5_*	5% mass loss of the sample;
*τ_10_*	5% mass loss of the sample;
ΔH	enthalpy of melting;
E′	storage modulus
E″	loss modulus;
E, MPa	young’s module;
σ_t_, MPa	tensile strength;
ε_b_, %	elongation at break;
T_g_	glass transition temperature;
T_m_	melting point;
tan δ	the tangent of the mechanical loss angle.
